# Draft Whole-Genome Sequences of Microbacterium oxydans and Microbacterium maritypicum Strains

**DOI:** 10.1128/mra.01089-22

**Published:** 2023-01-04

**Authors:** Nesrine Lenchi

**Affiliations:** a Department of Natural and Life Sciences, Faculty of Sciences, University of Algiers 1 Benyoucef Benkhedda, Algiers, Algeria; b Bioinformatics, Applied Microbiology and Biomolecules Laboratory, Faculty of Sciences, M'Hamed Bougara University of Boumerdès, Boumerdes, Algeria; University of Rochester School of Medicine and Dentistry

## Abstract

*Microbacterium* spp. are a group of microbes that have been recovered from a wide variety of environments in nature. Here, I report the complete genomic data for Microbacterium oxydans and Microbacterium maritypicum type strains that are already present in public culture repositories. The genome of the M. oxydans strain was 3,894,869 bp long, with a G+C content of 68.26%. The genome of the M. maritypicum strain was 3,668,377 bp long, with a G+C content of 68.44%.

## ANNOUNCEMENT

*Microbacterium* spp. are aerobic, Gram-stain-positive, rod-shaped bacteria. Widespread in nature, the genus *Microbacterium* comprises bacterial species relevant to humans, animals, plants, and the environment (1–3). In the present work, I report the whole-genome sequences of pure cultures of Microbacterium oxydans LMG 23389^T^ (= DSM 20578^T^), which was isolated from contaminated hospital material (4), and Microbacterium maritypicum LMG 8374^T^ (= DSM 12512^T^), which was isolated from seawater and marine mud (5), that were retrieved from the Belgian Coordinated Collections of Microorganisms (BCCM)/Laboratorium voor Microbiologie, University of Ghent (LMG).

Strains as dried material were cultivated and checked for purity after incubation at 28°C under aerobic conditions on Trypticase soy agar (TSA) (BBL 11768). Genomic DNA was isolated from isolated colonies using a Maxwell 16 tissue DNA purification kit (Promega) and a Maxwell 16 instrument (Promega) after a prior enzymatic lysis step with lysozyme (Sigma-Aldrich 62971) and mutanolysin (Sigma-Aldrich M9901). The integrity and purity of the DNA were evaluated with a 1.0% (wt/vol) agarose gel and spectrophotometric measurements at 234, 260, and 280 nm. A Quantus fluorimeter and a QuantiFluor ONE double-stranded DNA (dsDNA) system (Promega) were used to estimate the DNA concentrations.

Whole-genome sequencing (WGS) analyses were subsequently performed on each isolated strain. Accordingly, library preparation and WGS analyses of M. oxydans and M. maritypicum were performed by the Oxford Genomics Center (University of Oxford, Oxford, UK). Library preparation was performed using an adapted protocol for the NEBNext DNA preparation kit (New England BioLabs). Paired-end 150-bp sequence reads were generated using the NovaSeq 6000 platform (Illumina). Quality checks, trimming of the raw sequence reads, and *de novo* genome assembly were performed using the Shovill v0.9.0 pipeline (https://github.com/tseemann/shovill), which used SPAdes (6) as its core and aimed at a sequencing depth of 100×. Contigs shorter than 500 bp were excluded from the final assembly. Quality checks of the assemblies were performed using QUAST (7).

The assembled draft genomes of M. oxydans and M. maritypicum yielded 4 contigs (*N*_50_, 1,058,097 bp; *L*_50_, 2) and 5 contigs (*N*_50_, 2,227,735 bp; *L*_50_, 1), respectively. The genome of the M. oxydans strain was 3,894,869 bp long, with a G+C content of 68.26%. The genome of the M. maritypicum strain was 3,668,377 bp long, with a G+C content of 68.44%.

The draft genomes were then annotated using NCBI PGAP (https://www.ncbi.nlm.nih.gov/genome/annotation_prok), and secondary metabolites were predicted using antiSMASH v5.0.0 (https://antismash.secondarymetabolites.org) (8). Genome annotation by NCBI PGAP predicted 3,808 genes, including 3,751 coding sequences (CDSs) and 57 RNA genes (4 rRNAs, 50 tRNAs, and 3 noncoding RNAs [ncRNAs]), and 51 pseudogenes for the M. oxydans genome, whereas analyses of the M. maritypicum genome predicted 3,541 genes, including 3,488 CDSs and 53 RNA genes (3 rRNAs, 47 tRNAs, and 3 ncRNAs), and 36 pseudogenes.

Secondary metabolite cluster identification by antiSMASH predicted that the most similar cluster was the carotenoid biosynthetic gene cluster for both *Microbacterium* strains. In bacteria, carotenoids are involved in the mechanisms of membrane fluidity (9, 10) and protection of cells from oxidative damage and UV radiation (11, 12). In addition, both strains contain the ulleugmycine nonribosomal peptide synthetase (NRPS).

### Data availability.

The WGS shotgun projects have been deposited in DDBJ/ENA/GenBank under the accession numbers WAAP00000000 (BioProject accession number PRJNA573493) and WAAQ00000000 (BioProject accession number PRJNA573495) for Microbacterium oxydans and Microbacterium maritypicum, respectively. The raw reads have been deposited in the NCBI Sequence Read Archive (SRA) under the SRA accession numbers SRR21898868 and SRR21901517 for Microbacterium oxydans and Microbacterium maritypicum, respectively.

## References

[1] Gao M, Wang M, Zhang YC, Zou XL, Xie LQ, Hu HY, Xu J, Gao JL, Sun JG. 2013. *Microbacterium neimengense* sp. nov., isolated from the rhizosphere of maize. Int J Syst Evol Microbiol 63:236–240. doi:10.1099/ijs.0.038166-0. 22447697

[2] Kumari P, Bandyopadhyay S, Das SK. 2013. *Microbacterium oryzae* sp. nov., an actinobacterium isolated from rice field soil. Int J Syst Evol Microbiol 63:2442–2449. doi:10.1099/ijs.0.046870-0. 23203624

[3] Mondani L, Piette L, Christen R, Bachar D, Berthomieu C, Chapon V. 2013. *Microbacterium lemovicicum* sp. nov., a bacterium isolated from a natural uranium-rich soil. Int J Syst Evol Microbiol 63:2600–2606. doi:10.1099/ijs.0.048454-0. 23264499

[4] Schumann P, Rainey FA, Burghardt J, Stackebrandt E, Weiss N. 1999. Reclassification of *Brevibacterium oxydans* (Chatelain and Second 1966) as *Microbacterium oxydans* comb. nov. Int J Syst Bacteriol 49:175–177. doi:10.1099/00207713-49-1-175. 10028259

[5] Takeuchi M, Hatano K. 1998. Proposal of six new species in the genus *Microbacterium* and transfer of *Flavobacterium marinotypicum* ZoBell and Upham to the genus *Microbacterium* as *Microbacterium maritypicum* comb. nov. Int J Syst Bacteriol 48:973–982. doi:10.1099/00207713-48-3-973. 9734054

[6] Bankevich A, Nurk S, Antipov D, Gurevich AA, Dvorkin M, Kulikov AS, Lesin VM, Nikolenko SI, Pham S, Prjibelski AD, Pyshkin AV, Sirotkin AV, Vyahhi N, Tesler G, Alekseyev MA, Pevzner PA. 2012. SPAdes: a new genome assembly algorithm and its applications to single-cell sequencing. J Comput Biol 19:455–477. doi:10.1089/cmb.2012.0021. 22506599 PMC3342519

[7] Gurevich A, Saveliev V, Vyahhi N, Tesler G. 2013. QUAST: quality assessment tool for genome assemblies. Bioinformatics 29:1072–1075. doi:10.1093/bioinformatics/btt086. 23422339 PMC3624806

[8] Weber T, Blin K, Duddela S, Krug D, Kim HU, Bruccoleri R, Lee SY, Fischbach MA, Müller R, Wohlleben W, Breitling R, Takano E, Medema MH. 2015. antiSMASH 3.0: a comprehensive resource for the genome mining of biosynthetic gene clusters. Nucleic Acids Res 43:W237–W243. doi:10.1093/nar/gkv437. 25948579 PMC4489286

[9] Jagannadham MV, Chattopadhyay MK, Subbalakshmi C, Vairamani M, Narayanan K, Rao CM, Shivaji S. 2000. Carotenoids of an Antarctic psychrotolerant bacterium, *Sphingobacterium antarcticus*, and a mesophilic bacterium, *Sphingobacterium multivorum*. Arch Microbiol 173:418–424. doi:10.1007/s002030000163. 10896223

[10] Subczynski WK, Markowska E, Gruszecki WI, Sielewiesiuk J. 1992. Effects of polar carotenoids on dimyristoylphosphatidylcholine membranes: a spin-label study. Biochim Biophys Acta 1105:97–108. doi:10.1016/0005-2736(92)90167-k. 1314674

[11] Krinsky NI. 1978. Non-photosynthetic functions of carotenoids. Philos Trans R Soc Lond B Biol Sci 284:581–590.

[12] Miller NJ, Sampson J, Candeias LP, Bramley PM, Rice-Evans CA. 1996. Antioxidant activities of carotenes and xanthophylls. FEBS Lett 384:240–242. doi:10.1016/0014-5793(96)00323-7. 8617362

